# Improving the quality of counseling and clinical supervision in opioid treatment programs: how can technology help?

**DOI:** 10.1186/s13722-024-00435-z

**Published:** 2024-01-20

**Authors:** K. Michelle Peavy, Angela Klipsch, Christina S. Soma, Brian Pace, Zac E. Imel, Michael J. Tanana, Sean Soth, Esther Ricardo-Bulis, David C. Atkins

**Affiliations:** 1 PRISM, Department of Community and Behavioral Health, Elson S. Floyd College of Medicine, Washington State University, Spokane, WA USA; 2 Lyssn.Io, Seattle, Washington USA; 3 University of Utah, Salt Lake City, UT USA; 4 Evergreen Treatment Services, Seattle, Washington USA

**Keywords:** Opioid treatment, Addiction counseling, Motivational interviewing, Machine learning

## Abstract

**Background:**

The opioid epidemic has resulted in expanded substance use treatment services and strained the clinical workforce serving people with opioid use disorder. Focusing on evidence-based counseling practices like motivational interviewing may be of interest to counselors and their supervisors, but time-intensive adherence tasks like recording and feedback are aspirational in busy community-based opioid treatment programs. The need to improve and systematize clinical training and supervision might be addressed by the growing field of machine learning and natural language-based technology, which can promote counseling skill via self- and supervisor-monitoring of counseling session recordings.

**Methods:**

Counselors in an opioid treatment program were provided with an opportunity to use an artificial intelligence based, HIPAA compliant recording and supervision platform (Lyssn.io) to record counseling sessions. We then conducted four focus groups—two with counselors and two with supervisors—to understand the integration of technology with practice and supervision. Questions centered on the acceptability of the clinical supervision software and its potential in an OTP setting; we conducted a thematic coding of the responses.

**Results:**

The clinical supervision software was experienced by counselors and clinical supervisors as beneficial to counselor training, professional development, and clinical supervision. Focus group participants reported that the clinical supervision software could help counselors learn and improve motivational interviewing skills. Counselors said that using the technology highlights the value of counseling encounters (versus paperwork). Clinical supervisors noted that the clinical supervision software could help meet national clinical supervision guidelines and local requirements. Counselors and clinical supervisors alike talked about some of the potential challenges of requiring session recording.

**Conclusions:**

Implementing evidence-based counseling practices can help the population served in OTPs; another benefit of focusing on clinical skills is to emphasize and hold up counselors’ roles as worthy. Machine learning technology can have a positive impact on clinical practices among counselors and clinical supervisors in opioid treatment programs, settings whose clinical workforce continues to be challenged by the opioid epidemic. Using technology to focus on clinical skill building may enhance counselors’ and clinical supervisors’ overall experiences in their places of work.

The opioid epidemic has expanded substance use disorder (SUD) treatment services and forced rapid change in already stretched systems of care [1, 2]. In this climate, labor intensive implementation projects may be deprioritized, an unfortunate outcome if the implementation target is an evidence-based (EB) counseling practice that will ultimately help both patients and clinicians. Recording and reviewing counseling sessions is one notable barrier to implementation of EB counseling practices in community-based substance use disorder treatment [3], even though feedback and coaching are becoming the norm in Motivational Interviewing training research trials [4]. Concerns over the cost, time, and effort it takes to systematize procedures for recording counseling sessions are valid [5]. However, direct observation of clinical practice is not only imperative to arrive at EB fidelity [6, 7], it also fits the recommendation of SAMHSA’s Consensus Panel assembled to develop clinical supervision guidelines for SUD counselors [8].

Adequate clinical supervision is critical for EB implementation; it has also been demonstrated as a protective factor against burnout and prevention of turnover in SUD treatment settings [9–11]. Maintaining a healthy SUD workforce and the organizations in which they work is increasingly important as treatment providers hasten to accommodate a changing environment. From scaling up telehealth efforts in the context of COVID [12] to adapting treatment to an ever-evolving landscape of substances being used by SUD patients (e.g., methamphetamine and synthetic opioids; [13]), today’s SUD counselors face increasing pressures while they treat a more affected and abundant population than any other time in history. Such challenges highlight the need to support SUD counseling staff, both with hands-on supervision, as well as with tools that allow them to practice and feel confident about their clinical skills.

While technological innovation has been integrated into SUD treatment for the past several years, these innovations have not yet directly impacted clinical supervision and clinical skill building, including fidelity to evidence-based counseling practices. Thus far, technology efforts in the SUD treatment space have been limited to patient self-monitoring [14]; technology-based assessment, interventions and aftercare [15–17]; and mobile apps facilitating video to directly observe buprenorphine dosing for opioid use disorder [18]. Aside from web-based training for SUD counselors [19, 20], technology as a tool in SUD treatment has not yet been applied to the SUD workforce.

Recently, advances in machine learning and speech signal processing have been integrated into psychotherapy science, facilitating the development of technology to automatically evaluate use of specific evidence-based practices in recordings of substance use counseling and psychotherapy generally [21]. These machine-learning based approaches to fidelity monitoring, are typically trained by human labeled data (i.e., transcripts that have been coded with a gold standard fidelity measure like the Motivational Interviewing Skills Code [27]) and can be competitive with human to human reliability of the same sessions (see [22]). More recently, work has focused on initial testing of technologically based tools for fidelity monitoring with clinicians [23, 24]. However, there has been no formal study of barriers or facilitators to implementing technology supported supervision in real world SUD specific clinical milieus. Because clinical supervision can serve as a protective factor against burnout and turnover, it is important to examine innovative measures that promote quality and easy access to clinical supervision.

The purpose of this report is to first describe the uptake of a novel technology designed to increase SUD counselor skill in motivational interviewing and improve clinical supervision. Second, we aim to summarize results from focus groups targeting counselors and clinical supervisors in a large opioid treatment program, who discussed proposed implementation of a clinical supervision platform. Lyssn (or *Lyssn.io*) is a web-based platform that supports evidence-based supervision with machine learning based evaluation of motivational interviewing (MI; see [25]) skills in counseling sessions. This clinical supervision platform was designed with the intention of providing mental health providers session recording organization, immediate machine learning-based information about content, MI metrics, and a platform to facilitate supervision practices such as asynchronous discussion about the content of their sessions. The platform was developed with the use of machine learning to circumvent the use of laborious human coding techniques to provide session feedback (see [23]).

The current study examined implementation of a cloud-based recording and feedback platform in an Opioid Treatment Program (OTP), representative of treatment programs in the Pacific Northwest, as well as acceptability of this clinical supervision platform among counselors and clinical supervisors. We conducted two sets of focus groups (four total)—two with counselors and two with supervisors—from an opioid treatment program in Washington state. One set of counselor and supervisor focus groups were asked questions about current training and supervision practices. The second set were asked questions surrounding how this particular clinical supervision platform would help with clinical practice.

## Method

### Study site

The study site was a non-profit opioid treatment program (OTP) serving individuals with opioid use disorder across three geographically diverse locations in Washington State. Recruitment for the current study took place at the largest of the three sites, which serves Seattle’s urban core and contains upwards of 1400 patients. Many of these OTP patients (up to 60% in certain zip codes) are homeless, struggle with polysubstance use, as well as several psychosocial instabilities. As is consistent with SAMHSA guidelines for OTPs [26], counseling is mandatory at the study OTP; the treatment setup is such that counselors serve as the primary contact for OTP patients. Historically, the study OTP did not integrate direct observation (i.e., recording of counseling sessions) into clinical training or supervision. Unrelated to the study, the OTP was concurrently overhauling supervision protocols to include direct observation, and these changes are described in more detail in the focus group results.

The gold standard of treatment for opioid use disorder (OUD) is medication treatment (MOUD), involving one of the evidence-based medications targeting the disorder [46, 47]. However, there are several reasons to implement additional EBPs such as motivational interviewing in the context of MOUD [48, 49]. Adjunctive treatments may help increase adherence to treatments of infectious diseases that are common among MOUD patients (e.g., HIV, AIDS, hepatitis C), increase self-efficacy and engagement with MOUD, and assess for treatment readiness [50]. The site where the study took place provided MOUD via both methadone and buprenorphine to treat OUD. In addition, MI was utilized to aid with patient retention, MOUD treatment adherence, and targeting engagement with other health promoting behaviors (e.g., attending doctor’s appointments).

## Participants

Study site counselors (n = 27) and clinical supervisors (n = 2) were approached for inclusion in the current study at a counseling staff meeting, which served as the initial recruitment effort. New hires that occurred after initial recruitment were emailed about the opportunity to participate. While counselor participants represented a variety of educational and professional backgrounds, all participants had appropriate credentialing to practice SUD counseling in Washington State, entitled Substance Use Disorder Professional (SUDP) or a SUDP-Trainee designation for individuals on the pathway to licensure. Before this project, the majority of the counselors had no prior experience of recording and reviewing their clinical work.

Eleven participants and two clinical supervisors were recruited into the study and agreed to use the clinical supervision platform as a platform for recording counseling sessions with patients who provided consent for recording. Counselors and clinical supervisors then had the opportunity to participate in focus groups (germane to the current study), which were incentivized with $50 gift cards. Recordings of counselor and clinical supervisor focus groups were de-identified during the research process.

### Clinical supervision platform

**Fig. 1:**
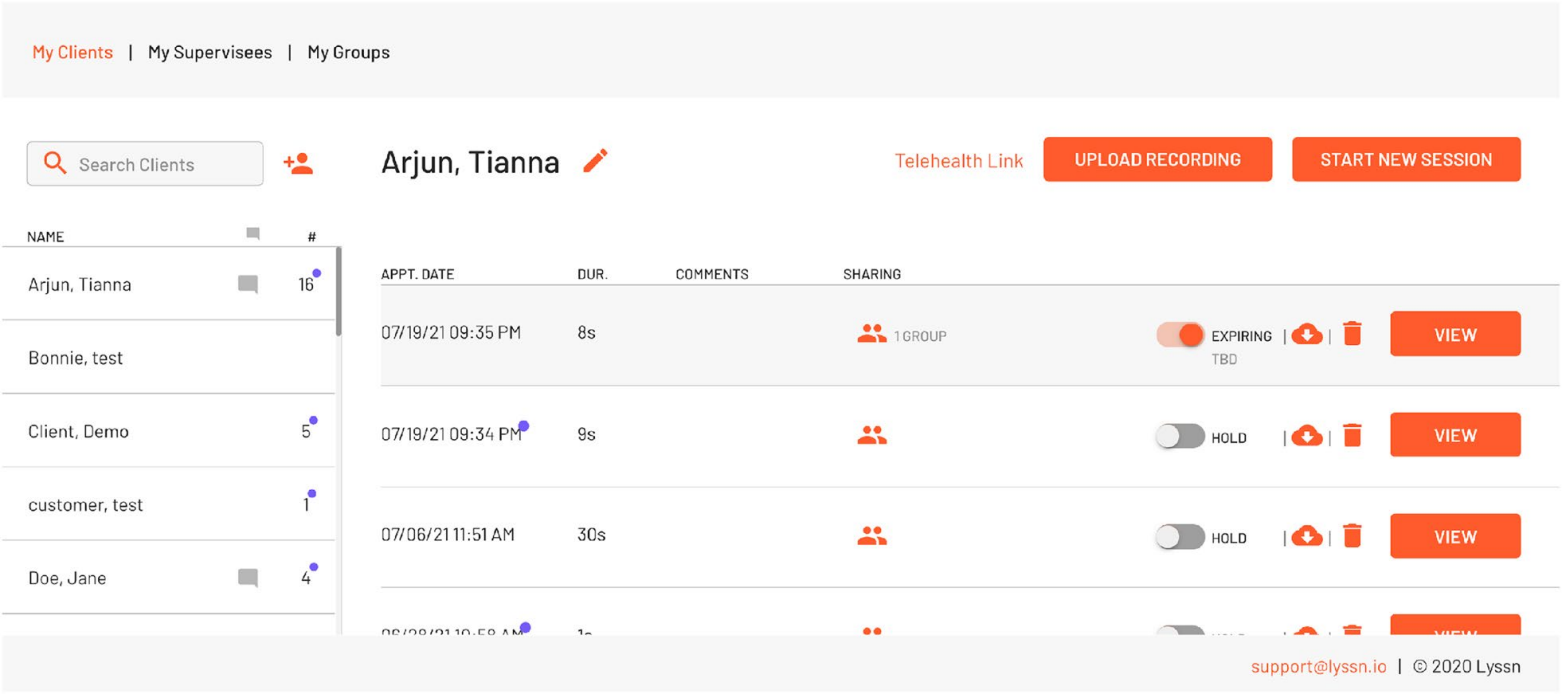
The Clincial Supervision Platform User Interface

**Fig. 2:**
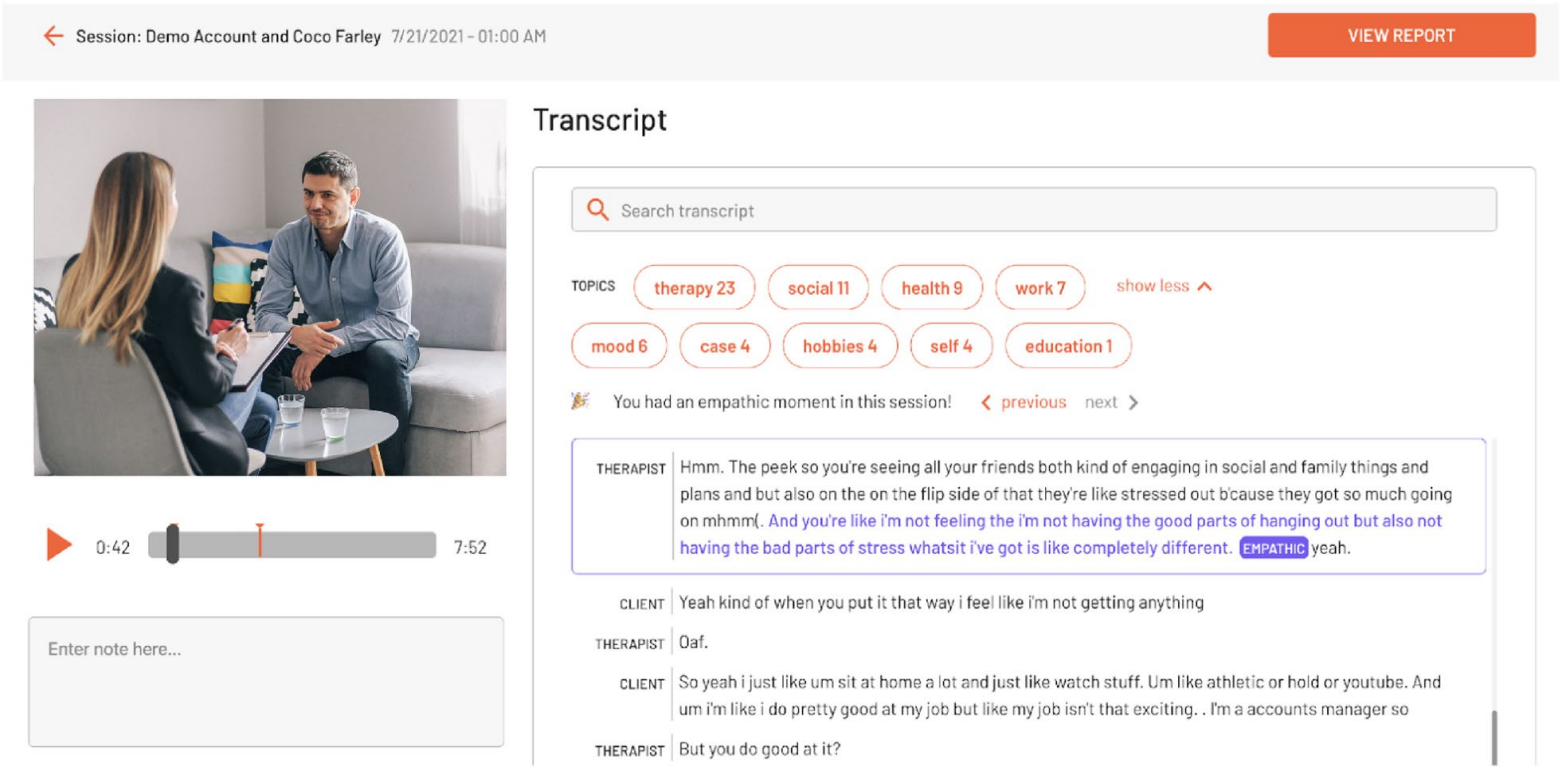
The Clinical Supervision Recorded Session Interface

**Fig. 3:**
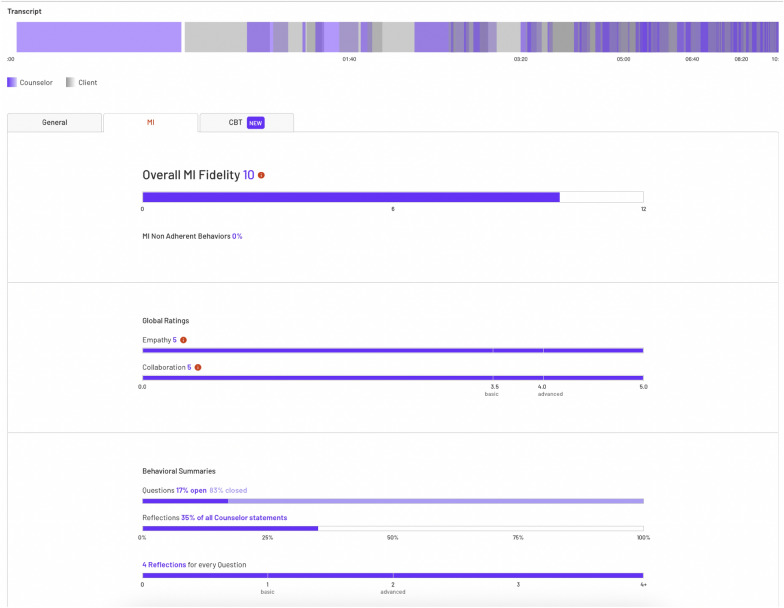
The Clinical Supervision MI Report User Interface

Prior to the focus groups, all participants were introduced to, and had the opportunity to use, the clinical supervision platform which provided recordings of counseling sessions, and machine learning based transcription, global MI metrics, and prevalent conversation topics. The focus group that was asked hypothetical questions about how the software could be used were additionally shown a report of the comprehensive machine-generated MI metrics (Fig. 3). Counselors and clinical supervisors accessed the clinical supervision platform via a web-browser, where users can easily record new counseling sessions or review previously recorded sessions (see Fig. 1). The session review interface supports two kinds of annotations: comments about the session as a whole in a simple chat box, as well as time-linked comments directly in the video playback (see Fig. 2). The latter allows clinicians and their supervisors to immediately queue up a portion of the session to review.

The cloud-based software included both a speech processing pipeline and a machine learning engine. The speech processing pipeline used state-of-the-art speech recognition algorithms, specifically designed for and trained on behavioral health conversations, to both generate a transcription, as well using speech features as inputs to predict the full suite of MI fidelity codes based on the Motivational Interviewing Skills Code system (MISC; [27]; see [23, 23, 41, 42, 44, 45]). In addition to continuing to improve the machine learning models from thousands of hours of human coded sessions, research has also been conducted to understand provider experiences in using the tool and making user-design informed changes (see [24]).

During the second focus group, concentrated primarily on hypothetical questions about how the platform could be used, counselors and clinical supervisors were introduced to an interactive, web-based report (Fig. 3), an additional feature. The report includes MI specific information via the traditional six MI fidelity statistics: empathy, MI spirit, reflection-to-question ratio, percent complex reflections, percent open questions, and percent MI adherent. In addition, there is a timeline of the entire session, where each talk-turn is linked to the automated speech recognition transcript of the session to facilitate review and study of specific exchanges within the session. Each talk-turn includes predicted MISC codes based on the machine learning engine and a visual representation of vocally encoded arousal of the speaker. In the focus groups, counselors and supervisors discussed how this feature could be integrated into clinical supervision and ultimately improve delivery of counseling services.

## Measures

To understand the acceptability of the clinical supervision software among counselors and clinical supervisors, four focus groups were conducted; two with counselors and two with clinical supervisors. Counselors and clinical supervisors were separated for each of the focus groups to encourage both groups to speak more freely about their experiences. The same questions were used for the counselor and clinical supervisor focus groups. Focus group #1 was used to: (1) gather general information about the current state of counselor training, clinical supervision practices, and monitoring; (2) briefly introduce staff to the clinical supervision software; and (3) discuss how the technology could be used in the current setting, and what the barriers would be. The objectives of Focus group #2 were to: (1) demonstrate the feedback report; and (2) discuss acceptability of the clinical supervision software including the feedback report, as well as motivators and barriers to implementation. Focus groups were conducted by members of the research team; the four focus groups were recorded and transcribed for analysis. In addition to focus groups, the number of sessions recorded through the clinical supervision software was also collected as a measure of engagement with the platform (see Table 1).

**Table 1:** Number of Recorded Sessions and Employment Turnover of OTP Counselors

Participant	Number of sessions recorded	Left employment (Y/N)
1	5	Y
2	8	N
3	23	Y
4	43	Y
5	9	N
6	91	N
7	71	N
8	0	Y
9	2	N
10	49	Y
11	125	Y

### Qualitative analysis

Participant responses during the focus groups were recorded and transcribed, and thematic analysis was employed to analyze transcripts. Focus groups with counselors included questions regarding how to use the computer-generated report, processes to review the report, using the report in supervision, and additional metrics that individuals would have liked to see in the report. For the clinical supervisor, there was an additional question of how counselors would react to using the software. For the analysis, two authors (KMP; BP) utilized a thematic analysis to identify common themes that emerged within the focus groups. The researchers then met and discussed emergent themes to reach a consensus, resulting in the conceptual categories illustrated by participants' comments. Researchers met regularly to resolve disagreements on conflicting theme coding, discuss their rationale for codes, and share their personal biases that may have influenced the coding process.

## Results

Table 1 summarizes information about 11 counselors who agreed to participate in the study, how many sessions they recorded. A total of 426 sessions (*M* = 38.7; *SD* = 41.7; range = 0–125) were recorded over a 9-month period. Counselors conduct approximately 80 sessions per month, so there were approximately 7,920 number of sessions possible. Patients need to consent before being recorded, which may have limited the overall number of possible sessions. We did not collect the number of patients who consented to record.

### Focus group results

#### Theme 1: The clinical supervision software as a mechanism for focusing on counseling and clinical supervision as opposed to documentation

A perceived benefit of the clinical supervision software was that it is a mechanism for focusing on counseling skills, as opposed to administrative and documentation requirements. One counselor said: “I would like to know what I could do differently to elicit a different response, to show more empathy. To really learn that. With chemical dependency there’s a lot of case management all of that. I’d like to actually have a counseling session.” Another counselor indicated: “I know that ‘counselor’ is attached to our names, but it’s like we’re paper pushers. And we do a lot of paperwork, and we have to do a lot of paperwork by a certain amount of time and a certain set of paperwork. And that’s our focus. It’s not really like eliciting this change talk within the patient. You know that’s kind of gone way out here for me. I want to know how I could do it differently.”

#### Theme 2: The clinical supervision software as an aid to supervision

Counselors were largely very positive about their experience in clinical supervision; they described their Clinical Supervisor as supportive and empowering. “[Clinical Supervisor] always makes time for you. When I leave [Clinical Supervisor’s] office I feel like a superhero. Like I could take on the world. When you leave the office to go downstairs, it’s important to have 110%.” The process for obtaining Clinical Supervision was described as both informal (e.g., “I just knock on [Clinical Supervisor’s] door”), as well as formal, scheduled individual and group supervision. Supervision reportedly consists of clinical consultation along with discussion of processes/policies and documentation review. Clinical Supervisors indicate that the current and primary mechanism for determining counselor performance depends heavily on documentation review and has little to no progress reporting. “If there’s a tool that’s going to make tracking and helping…because now I’m going to have to be doing…it’s almost like progress notes with on the people I supervise which I’ve not had to do in the past unless it was like a corrective action kind of plan. Uh so that’s going to be new and if this kind of technology is going to help with that I’m definitely all for it.”

Both counselors and clinical supervisors talked about the value of ongoing formal training (i.e., didactic; Continuing Education), specifically citing motivational interviewing as a primary interest. “Training is so important…it’s so empowering to the whole idea of counseling. You need it. It would be really nice if I felt like I had the time to leave [for training]. This clinic is just a little busy.” Counselors and clinical supervisors cite large caseloads and increasing responsibilities as a barrier to not only training attendance, but also as a barrier for targeted skill development. Clinical supervisors noted that “one-off” trainings were important, but such a format had limitations in terms of producing substantive skill improvement across the counseling staff. One supervisor remarked that “…you get four, 5 days or a week’s worth of that training, but then there needs to be follow-up. How do you implement it?”.

Clinical Supervisors voiced excitement about how the clinical supervision software will help implement the new supervision protocol. To maintain consistency with SAMHSA’s Clinical Supervision Treatment Improvement Protocol TIP 52 [8], supervisors reported that the clinical supervision software’s recording platform could help them meet this objective more efficiently than sitting in sessions with counselors. “… [Observation] is probably part of the new managed care, value-based care, kind of uh model that we’re all uh getting prepared to deal with.” They reported that the clinical supervision software feedback forms could help supervisors formulate their own evaluative commentary more quickly, noting that simultaneously observing counseling and writing feedback is “time consuming.” Another piece of the new supervision protocol that the clinical supervision software could help with is the supervision documentation that will be an expectation with the new protocol. One supervisor reported: “It’s almost like [we need to keep] progress notes for supervision—that’s new. If this kind of technology would help with that, I’m all for it.”

Supervisors noted that the clinical supervision software’s recording platform and feedback form could help shape the content of supervision sessions themselves: “I’m a little bit nervous about…when it comes time to doing direct observation…kind of knowing okay, what should I be focusing in on? So I can give feedback that’s going to be useful.” Supervisors see how the clinical supervision software feedback report could help structure counselor feedback and help them tailor supervision based on skills being displayed, or not displayed, according to the report.

Supervisors reported that session recordings could ensure good clinical care. “[Recording sessions] is how you know you’re providing the good service…You look at the note, you know, and that tells you they can document. Doesn’t tell you what kind of counselor they are per se.” Supervisors noted the feedback is easier to give to counselors who are performing well, and more difficult to give then counselors who are not performing well. They make the point that the clinical supervision software’s feedback form could pull out objective information, making the process easier to give feedback to individuals who are not performing well.

#### Theme 3: Session transcription could improve counselor workflow

Counselors were excited by the transcription function of the clinical supervision software, seeing this feature as both a way to help them chart more efficiently, and as a means for connecting more genuinely with patients. One counselor noted: “When you’re doing counseling you’re either sitting at a computer and typing or you’re taking handwritten notes, or you commit what they say to memory. I’d rather not be doing any three of those things when I’m working with a patient. So ultimately I would use the transcript to help complete my notes.”

#### Theme 4: Feedback as a tool to improve counselor MI skills

Counselors expressed an interest in how the clinical supervision software’s feedback could help them improve their clinical skills. Said one counselor: “Just having a reflective practice about how I am doing as a counselor. My whole generation of friends, we’re all gamers right? So having a score to compare myself to. It’s something to challenge myself to. To improve on.” Counselors also considered how the immediacy of the feedback forms could transfer to skill uptake. For example: “I don’t want to wait three times a year for a supervisor to come in and tell me how I’m doing on my OARS skills, I want to know more frequently than that. This is why I signed up for this project in the first place.”

#### Theme 5: Concerns about recording

Counselors made comments throughout the focus groups about work capacity, citing high caseloads and limited time to take on new tasks. This issue was top of mind for counseling staff, and one concern with the clinical supervision software’s implementation was that it would be adding to an existing full plate of responsibilities. They noted adding in a process to their routines can be difficult to integrate and remember (one counselor noted that they had a hard time remembering to start recording). Another person remarked that adding in another consent for patients may seem overwhelming to them, given that patients have an existing set of consents they sign at the outset of treatment.

Counselors expressed concerns about how recording might affect clinical interactions and patient experiences. Specifically, there was a mixture of responses about counselors’ experiences in getting patient consent to record: “My population is not very trusting of systems. They‘re like ‘Wait wait, you want to record this for what?’ And it takes a lot of conversation and some of them are still just resistant.” Another counselor also expressed initial concerns about the patients’ willingness to record but observed patients’ overall desire to be helpful to the counselor, their growth, and in turn helpful to fellow patients: “For the patients, for the most part, when I talk to them [about recording counseling] they seem to be open. ‘If it helps somebody behind me I will do it’. That sense of goodwill. I wasn’t expecting that.” Consensus was that consent to record depended on both the patient variables (e.g., whether patients present with paranoid ideation), as well as how the opportunity is presented (i.e., emphasis of recording is on counselor, and as a way to help counselor skills, as opposed to focus on patient content).

Counselor’s experiences with recording varied based on prior recording experiences. One counselor who had recorded sessions states: “I really hesitated to get [involved with] this project because I wasn’t too sure…at what part…how genuine the patient would be in the session. Because you have the recording right there. And how genuine is the patient? But as it progressed I didn’t think that was an issue. And that was…it was a really big relief. If anything else it put me in a place it made it a lot easier for me to focus. Into the session.” Finally, counselors noted a concern about security, both in terms of outsiders accessing recordings, as well as the possibility of recordings being subpoenaed by law enforcement or Child Welfare organizations.

Like counselors, supervisors also predicted time constraints to be a barrier to the clinical supervision software implementation. One supervisor put it: “The thought of adding anything new, even if it’s positive, can be a barrier.” Supervisors indicated that direct observation via recorded counseling sessions would be a culture shift among counseling staff, and they wondered whether counselors would be mistrustful of this level of oversight. “Maybe there would be fear that I would judge them, or that they would get in trouble.” They noted that openness to this level of supervision would likely vary based on counselor variables; one Clinical Supervisor speculated that newer counselors may be more open to it. “They are learning and absorbing.”

## Discussion

The current study tested the acceptability of the clinical supervision software, a recording platform for counseling sessions that provides transcripts, as well as an AI-backed report that provides counselors and supervisors real time feedback about counselor performance and MI skills. At the study site, a large number of counselors opted to try out the new technology, albeit a number had left the OTP during the clinical supervision software trial period, indicative of high turnover found in OTP sites [28]. Overall, after reviewing two sets of focus groups (four sessions total), we found that the clinical supervision software was experienced by counselors and clinical supervisors as beneficial to counselor training, professional development, clinical supervision, and importantly to the provision of counseling to OTP patients.

Our data indicate that 11 counselors at a large OTP voluntarily tried the clinical supervision software to improve their skills; these counselors recorded a total of 426 counseling sessions over 9 months demonstrating uptake of a new technology. An organization needs early adopters to help bring their peers along, and the counselors who participated in the current study serve as a proxy for “champions”, a key piece to successful implementation of innovations in SUD treatment settings [29].

Supervisors and counselors alike expressed enthusiasm about the clinical supervision software and its potential impact on clinical supervision, albeit for slightly different reasons. Counselors reported that they value clinical supervision, describing it as supportive and vital to their work. This sentiment is common amongst SUD counselors [30], but supervision practices vary greatly [31], one study indicating that up to a third of counselors did not even receive any clinical supervision [32]. Other researchers have shown that supervisors in addiction treatment settings tend to report more time provided to supervision, more interactions and feedback than do their matched counselor counterparts [33]. A clinical supervision software tool could have the effect of prompting consistently scheduled supervision that has a purpose and structure.

Study site supervisors noted that the clinical supervision software could aid with the increasing supervision demands from state and national agencies, as well as the added supervision documentation included in these new requirements. Specifically, supervisors reported that their organization was already moving towards requiring a more codified and systematic clinical supervision protocol. Interviews with SUD treatment providers about value-based care indicate concerns about staff not being adequately trained in evidence-based practices, and that the training burden would be challenging [34].

While counselors were not as closely attuned with forthcoming supervision protocols and quality assurance issues, they did express interest in getting immediate and more frequent feedback about their performance. Feedback from counselors has since informed the development of an automatically generated session summary that is now provided by the clinical supervision software as another way to aid with the clinical documentation process. Though participants noted concern regarding security and the potential for recordings to be subpoenaed, they posited that the use of a recording platform to deepen their understanding of session content was highly valuable. Future implementation efforts should continue to focus on ensuring the privacy, security, and parameters in which recordings may become part of a court proceeding, with the ultimate goal of protecting providers and their patients.

Supervisors remarked that the clinical supervision software’s machine generated feedback reports would assist in focusing on skills and allow more accurate and objective feedback about clinical skills. Supervisors reported that the organization currently depends on documentation review to gauge performance. Recording and feedback reports would take some of the guesswork out of performance evaluation. Counselors may also appreciate more objectivity in measuring performance, which could ultimately lower turnover. Clinical supervisors also noted that one-time trainings, though important, lacked the necessary follow up that research has demonstrated to be necessary for skill retention [35]. If routinely used in clinical supervision, the software may be perceived by counselors that feedback is more objective, obviating quality of care to both counselor and clinical supervisor, taking favoritism (or perceptions of such) out of the running as a driver of performance evaluation results.

Counselors and supervisors saw the clinical supervision software as a tool to help counselors improve their Motivational Interviewing skills by regularly listening to counseling sessions, reviewing the feedback form, and using these tools to reflect on their own work. Counselors also remarked that the clinical supervision software commands a real focus on clinical skills, an emphasis that is a change of pace given that they feel their jobs are consumed with “paper pushing” activities. In other words, counselors spoke to a possible conflict between their values (e.g., counseling; rapport building) and the values of the larger organization and regulating bodies (e.g., documentation and other requirements that detract from patient/counselor interactions). These types of value conflicts have been shown to play a role in burnout and turnover [36]. At any job there is a balance between the work employees must do and what they want to do. When asked in a qualitative study, rural SUD counselors noted that increased access to professional education and opportunities would enhance recruitment and retention in the workplace [37]. An AI-based supervision platform could aid counselors by auto summarizing chart notes and keep counseling skill development top of mind, the balance between “have tos” and “want tos” is weighted in a way that counselors’ work can remain connected to the reasons many of them were drawn to the field in the first place: because they wanted to help.

Like many large and complicated OTPs, counselors and supervisors stated that they had a great number of existing demands, making the implementation of anything new more challenging. Supervisors also wondered about the acceptance of the clinical supervision software more broadly, understandably considering counselors who did not volunteer to participate. They acknowledged that with a heterogeneous staff it makes sense that some will be open to it and others not. Reluctance to record work samples or have work directly observed is commonplace among SUD providers [32]. Changing cultural organization to promote consistent recording may indeed appear as a sizable obstacle; however, direct observation is not only what is recommended in SAMHSA TIP 54, but also shown to be more accurate and comprehensive in terms of understanding what counselors are doing with their patients [38].

## Limitations

Though presenting powerful results that represent some of the realistic challenges for counselors and clinical supervisors, there are some limitations to the study. Counselors who volunteered for participation in the study may have been more eager and enthusiastic to participate, and therefore results may be difficult to generalize. The counselors that volunteered for the study likely represent those that tend to show more openness to new learning opportunities and perhaps more motivated to improve their work.

In a post-hoc collection of counselor attrition data, of the 11 counselors recruited into the study, more than 50% (*n* = 6) left employment during the pilot period. Due to turnover in counseling staff, there was heterogeneity in counselor experience and exposure to the technology. Some focus group participants had substantial experience with the clinical supervision software, others had not used it at the time of the focus group. SUD counselors continue to face new and increasing issues that lead to burnout and high turnover [39]. As with any stretched system, SUD treatment organizations may at best prioritize immediate crises and other pressing tasks over EB counseling implementation, at worst skimp on clinically vital activities such as supervision. The COVID-19 pandemic continues to contribute to significant workflow disruptions, implicating long standing changes to OUD treatment [40], and ushering in widespread reliance on technology (i.e., telehealth; [12]). New systems and telehealth practices may provide opportunities for patients and providers to remain connected, however, these systems continue to add some burdens to counselor workflows and prohibit some counselors from consistently using new technology.

Finally, the sample of participants who volunteered to participate in focus groups was small, and pulled from one treatment setting, and thus also difficult to generalize to the broader network of OTP counselors and clinical supervisors. Further research should be conducted with OTP clinics to help generalize the benefits and continued needs of these mental health providers.

## Conclusion

Technology can play a positive role in supporting the implementation of evidence-based counseling at sites like an OTP, where workloads are stretched due to the ongoing opioid epidemic. Counselors and clinical supervisors interviewed in our focus groups were enthusiastic about the clinical supervision platform’s utility in improving motivational interviewing skills and enhancing clinical supervision. To support the SUD workforce, we need to find innovative ways to help clinicians feel connected to their work and confident in the clinical supervision they receive. Treatment settings and researchers should continue to consider how technology can improve the services provided to people with SUDs.

## Data Availability

Data sharing is not applicable to this article as no datasets were generated or analyzed during the current study.
